# Stokes' Second Problem for Magnetohydrodynamics Flow in a Burgers' Fluid: The Cases γ = λ^2^/4 and γ>λ^2^/4

**DOI:** 10.1371/journal.pone.0061531

**Published:** 2013-05-08

**Authors:** Ilyas Khan, Farhad Ali, Sharidan Shafie

**Affiliations:** Department of Mathematical Sciences, Faculty of Science, Universiti Teknologi Malaysia, Skudai, Malaysia; Rensselaer Polytechnic Institute, United States of America

## Abstract

The present work is concerned with exact solutions of Stokes second problem for magnetohydrodynamics (MHD) flow of a Burgers' fluid. The fluid over a flat plate is assumed to be electrically conducting in the presence of a uniform magnetic field applied in outward transverse direction to the flow. The equations governing the flow are modeled and then solved using the Laplace transform technique. The expressions of velocity field and tangential stress are developed when the relaxation time satisfies the condition γ = λ^2^/4 or γ>λ^2^/4. The obtained closed form solutions are presented in the form of simple or multiple integrals in terms of Bessel functions and terms with only Bessel functions. The numerical integration is performed and the graphical results are displayed for the involved flow parameters. It is found that the velocity decreases whereas the shear stress increases when the Hartmann number is increased. The solutions corresponding to the Stokes' first problem for hydrodynamic Burgers' fluids are obtained as limiting cases of the present solutions. Similar solutions for Stokes' second problem of hydrodynamic Burgers' fluids and those for Newtonian and Oldroyd-B fluids can also be obtained as limiting cases of these solutions.

## Introduction

Magnetohydrodynamics is the study of flow of electrically conducting fluids in electric and magnetic fields. This phenomenon is essentially one of the mutual interaction between the fluid velocity and electromagnetic field i.e. the motion of the fluid affects the magnetic field and the magnetic field affects the fluid motion. Basically, magnetohydrodynamics is a research area that involves the study of motion of electrically conducting fluids such as plasma and salt water. MHD flows are found to have influential applications in many natural and man made flows. They are frequently used in industry to heat, pump, stir and levitate liquid metals. Another application for MHD is the magnetohydrodynamic generator in which electrically conducting fluid is used to generate electric power. The flows of an electrically conducting fluid in the presence of a magnetic field have important applications in various areas of technology such as, accelerators centrifugal separation of solid from fluid, purification of crude oils, astrophysical flows, petroleum industry, polymer technology, solar power technology, nuclear engineering applications and other industrial areas [1], [2].

The literature on the study of MHD viscous fluid is abundant (see for example [3]-[10] and the references therein). However, such studies for non-Newtonian fluids are limited. To the best of author's knowledge, MHD flow of non-Newtonian fluids was first studied by Sarpkaya [11]. Subsequently, several other investigations considering the MHD flow of non-Newtonian fluids were carried out and currently this field has become an active area of research. Ersoy [12] examined the MHD flow between eccentric rotating disks for an Oldroyd-B fluid. Hayat and Hutter [13] obtained exact solutions for flows of an electrically conducting Oldroyd-B fluid over an infinite oscillatory plate in the presence of a transverse magnetic field. Khan et al [14] developed exact solutions of Stokes second problem for MHD Oldroyd-B fluid. Liu et al [15] and Zheng et al [16] and [17] analyzed the MHD flow of generalized Oldroyd-B fluid for different fluid motions using frictional derivatives. On the other hand, studies on MHD flow of Burgers' fluid are very limited. Therefore, any MHD analysis of this model will be genuine contribution towards the enhancement of the theory of non-Newtonian fluid mechanics. Hayat et al. [18] studied the MHD flow of Burger's fluid whereas with heat transfer analysis was investigated by Siddiqui et al. [19], [20]. Very recently, Khan et al [21] studied MHD flow of Burger's fluid and obtained exact solutions of Stokes' first problem by using the Laplace and Fourier sine transforms. The MHD flows of these fluid models and some other well known non-Newtonian fluids models such as second grade fluid [22]–[27], third grade fluid [28], Maxwell fluid [29], [30], generalized Burgers' fluid [31], [32], Micropolar fluid [33], [34], Walters-B liquid fluid [35], Jeffery fluid [36] and Nanofluid [37] are used to describe stress relaxation, shear thinning or shear thickening, normal stress effects, earth's mantle, asphalt and asphalt mixes, food products and soil, dilute polymeric solutions, hydrocarbons, paints and several other industrial and geomechanical fluids.

Khan et al [38] extended the work of Fetecau et al [39] to the MHD flow of an Oldroyd-B fluid induced by the impulsive motion of a plate between two side walls perpendicular to the plate. The analytical solutions are carried out by using the Fourier sine and Laplace transforms. Vieru et al [40] determined exact solutions corresponding to the flow of a Burgers' fluid over a suddenly moved flat plate when the relaxation times satisfy the condition 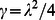 or 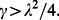 They used the Laplace transform technique to find the expressions for velocity and shear stress fields which were reduced to the similar solutions for Newtonian and Oldroyd-B fluids as limiting cases. Recently, Khan et al [41] extended the work of Vieru et al [40] to the flow of a Burgers' fluid over an oscillatory moved flat plate. They used a similar method of solution and obtained the exact solutions.

From the literature survey, it is found that there are very few problems of Newtonian fluids for which the exact solutions are available. However, these solutions become even more rare if the constitutive equations of non-Newtonian fluids are considered. The importance of exact solutions is not only that they can explain the physics of some fundamental flows but also that such solutions can be used as checks against complicated numerical codes that have been developed for much more complex flows. Moreover, one of the most common mistakes that has been overlooked for the last coupled of decades has been identified by Christov [42]. Christov pointed out that in the case of Stokes first and second problems, the plate's velocity is given by 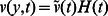, where 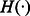 denotes the Heaviside step function, and 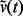 is some smooth function. This inclusion of Heaviside step function was ignored previously. There are several comments and errata published in the literature for the modification of such erroneous results. It is important to mention here that such type of mistakes reported by Christov [42] are avoided in the present communication.

The main purpose of the present investigation is to extend the work of Vieru et al. [40] and Khan et al. [41] for the MHD flow of an electrically conducting Burgers' fluid past an oscillating plate when the magnetic field is acting perpendicular to the flow direction. It is also interesting to study the flow of non- Newtonian fluids with externally imposed magnetic fields which control the boundary layer and increase the performance of many systems. For example, when we use the electrically conducting fluid in MHD power generators, their performance increase in comparison to conventional electric generators where solid conductors are used to generate electric power. The present work can also be helpful to study underground oil, where there is a natural magnetic field and the motion of blood through arteries [43], [44].

The rest of the paper is arranged as follows. The governing equations of the problem are given in section 2. The mathematical formulation of the problem is given in Section 3.The solution of the problem is given in section 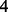
where the Laplace transform technique is used and the expressions for velocity and shear stress fields are obtained when the relaxation time satisfies the condition 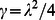 or 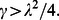
Limiting solutions are given in section 5. Graphical results are displayed in section 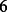
and discussed for the embedded flow parameters. This paper ends with some conclusions given in section 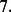


## Governing Equations

The unsteady incompressible flow of an electrically conducting fluid is governed by the following equations
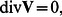
(1)

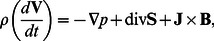
(2)

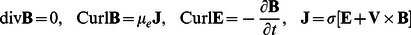
(3)where 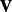
is the velocity vector, 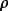
is the density of the fluid, 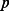
is the pressure,
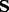
is the the extra stress tensor, 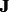
is the current density, 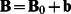
is the total magnetic field where 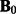
denotes the applied magnetic field and 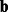
is the induced magnetic field, 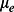
is the magnetic permeability, 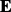
is the electric field and 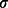
is the electrical conductivity of the fluid.

The extra stress tensor 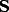
for non-Newtonian Burgers' fluid constitutes the following equation [40], [41]

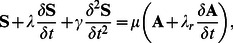
(4)in which
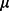
is the dynamic viscosity,
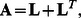
is the first Rivlin Ericksen tensor, 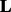
is the velocity gradient,
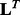
is the transpose of the velocity gradient,
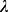
and
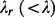
are the relaxation and retardation times respectively and 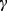
is the material constant of Burgers' fluid multiplies the upper second order convected time derivative of 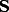
defined as
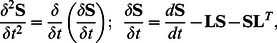
(5)where 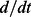
is the material time derivative.

For the problem under consideration, we are looking for velocity and stress fields of the form
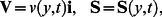
(6)where 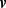
is the 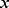-component of velocity field 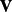
and 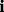
is the unit vector in the 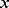-direction.

In order to calculate Lorentz force, it is assumed that the polarization effects are zero (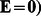, the magnetic field 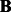
is applied in outward perpendicular direction to the flow and the induced magnetic field 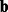
is negligible compare to the applied magnetic field 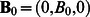
under the assumption of small magnetic Reynolds number, 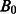
is the strength of applied magnetic field. Thus in view of these assumptions and using Eq. (3), the Lorentz force becomes [21]

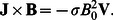
(7)


Thus using Eq. (6), the continuity Eq. 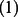
is identically satisfied and the momentum Eq. (2) in the absence of a pressure gradient in the flow direction and Eq. (4) after using Eqs. 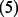
and (7) and having in mind the initial conditions 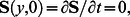
give the following governing equations
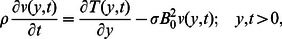
(8)

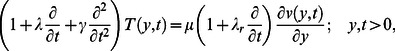
(9)where 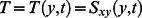
is the non-trivial shear stress.

## Mathematical Formulation of the Problem

**Figure 1:**
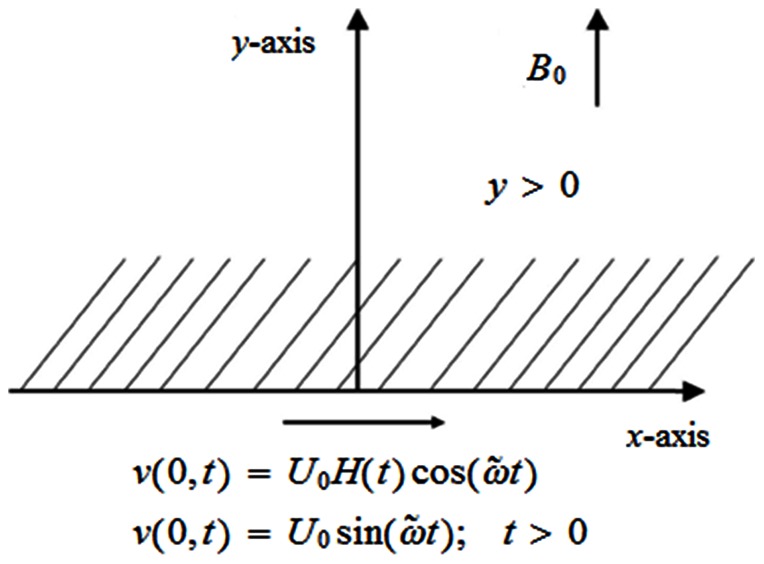
Physical model and coordinates system.

We consider the unsteady incompressible flow of an electrically conducting Burgers' fluid occupying the upper half space of 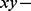
plane over a rigid flat plate. The 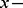
axis is taken parallel to the flow direction whereas 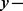
axis is taken normal to the plate. The magnetic field is applied in outward transverse direction to the flow. Initially, we assume that both fluid and plate are at rest. After time 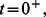
the plate begins to oscillate in its own plane and the fluid is gradually moved as shown in Fig. 1.

For such type of motions the governing equations are (8) and (9) with the following initial and boundary conditions
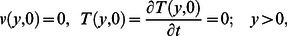
(10)

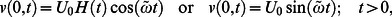
(11)where 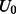
is the characteristic velocity, 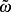
is the imposed frequency of the velocity of the plate and 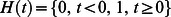
is the Heaviside step function.

Moreover, the natural conditions 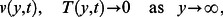
(12)which are the consequences of the fact that the fluid is at rest at infinity and there is no shear in the free stream, have to be also satisfied.

## Solution of the Problem

Introducing the following non-dimensional variables
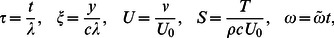
(13)with the constant 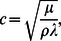
the governing Eqs. 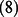 and 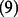
take the following forms
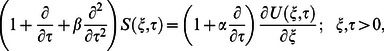
(14)

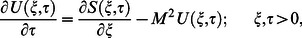
(15)where
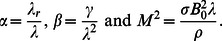



The corresponding initial and boundary conditions 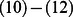
become
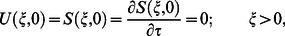
(16)

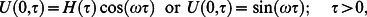
(17)

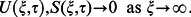
(18)


In order to solve the initial and boundary-value problem 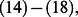
we consider two different cases 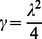
and 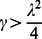
and use the Laplace transform.

**Case-I: Solution of the problem for** 
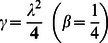


In order to determine exact solutions for our problem, we substitute 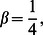
into Eq. 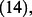
apply the Laplace transform to Eqs. 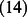
and 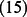
and use the initial conditions 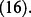
We find that
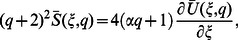
(19)

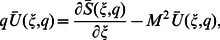
(20)where 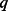
is the transform parameter. In view of the boundary conditions, the Laplace transforms 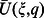
and 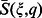
of 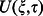
and 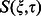
have to satisfy the conditions



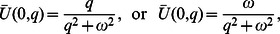


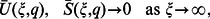
(21)where



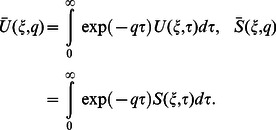
(22)The solutions of Eqs. 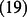
and 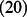
satisfying the boundary conditions 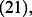
are
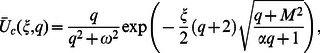
(23)

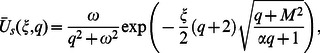
(24)

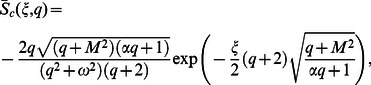
(25)

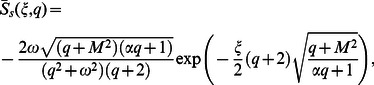
(26)where the subscripts 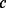
and 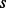
denote the solutions corresponding to the cosine and sine oscillations of the boundary, respectively.

In order to find 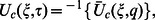
we follow a similar procedure to 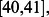
and write Eq. 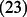
in the product form
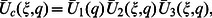
(27)where



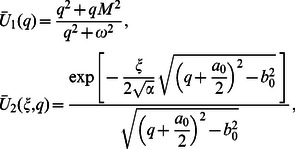
(28)

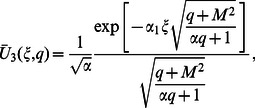
(29)

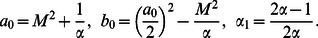
(30)


Of course, in view of Eq. 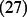
we have
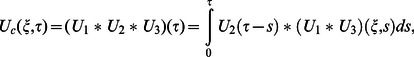
(31)where the denotes the convolution product and 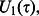 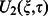
and
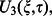
are the inverse Laplace transforms of 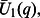 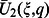
and 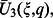
respectively.

Applying the inverse Laplace transform to Eqs. 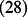
and 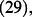
we find that
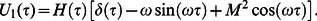
(32)

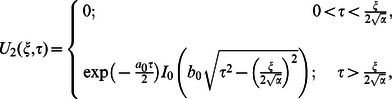
(33)

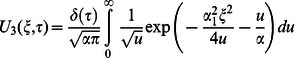


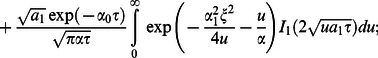
(34)

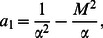
(1)where 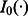 and 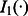
are the modified Bessel functions of the first kind of order zero and order one respectively.

Now using Eqs. 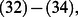
into Eq. 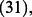
and by the definition of Heaviside step function, we get
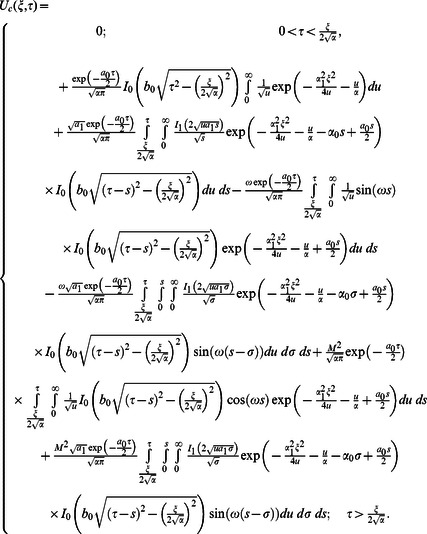
(35)


Similarly for the sine part of velocity, we get the following expression
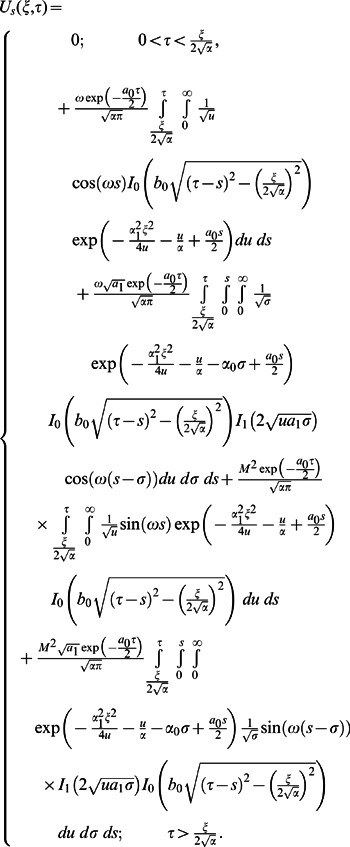
(36)


In order to find the dimensionless shear stress, we write 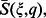
given by Eq. 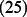
in the form
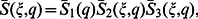
(37)where



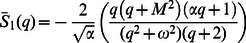
(38)

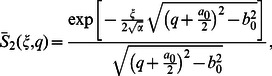
(39)

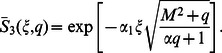
(40)


For 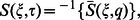
we employ
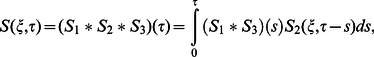
(41)


with
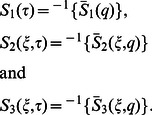
(42)


The Laplace inverse transforms of Eqs. 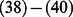
yields
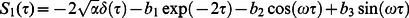
(43)

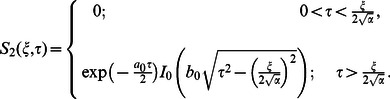
(44)

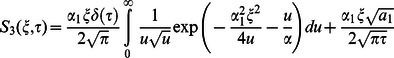


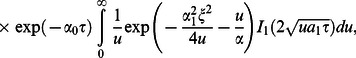
(45)where



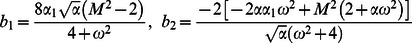


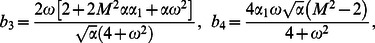


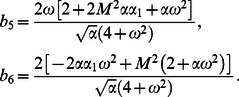
(46)


The convolution product of Eqs. 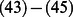
gives
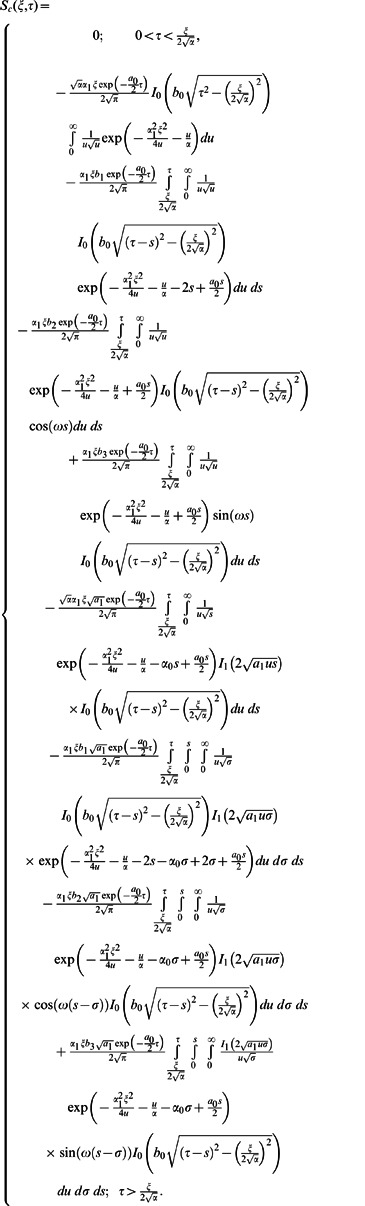
(47)


Similarly for sine oscillation we obtain
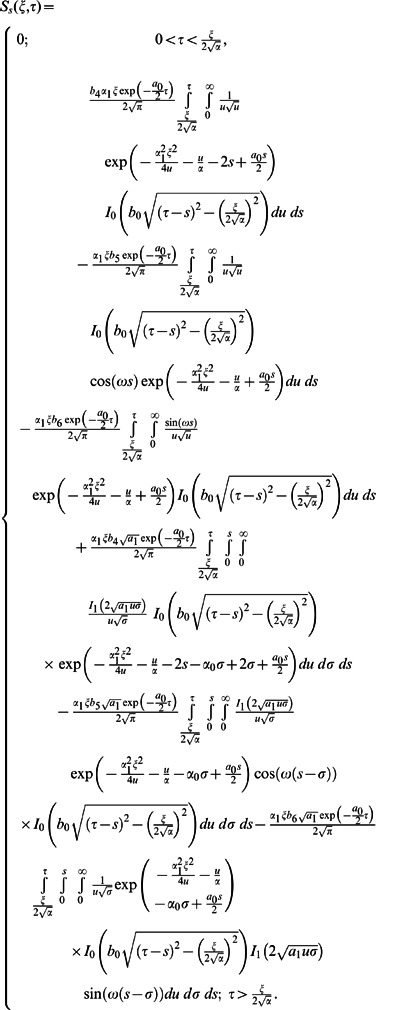
(48)


Furthermore, it is noted that the expressions 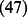
and 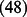
are valid only for 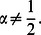
Therefore, we are separately considering the case when 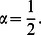
Hence, Eq. (25) can successively be written in the form
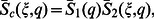
(49)where



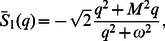
(50)

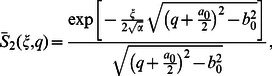
(51)

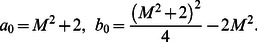
(52)


The inverse Laplace transforms of Eqs. 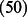
and 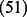
are given as
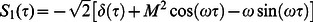
(53)

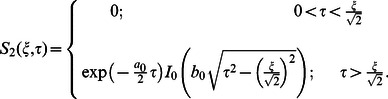
(54)


Now taking the convolution product of Eqs. 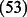
and 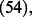
we finally obtain
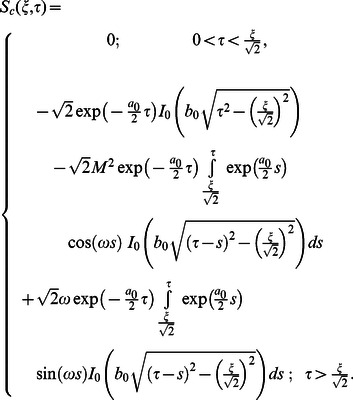
(55)


In the same way we find that
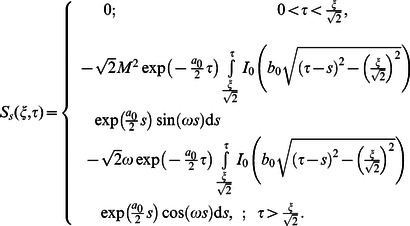
(56)


Now, in order to find the associated expressions for velocity, we directly put 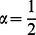
into Eqs. 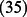
and 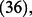
make the change of variable 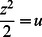
in the first integral, and finally we get
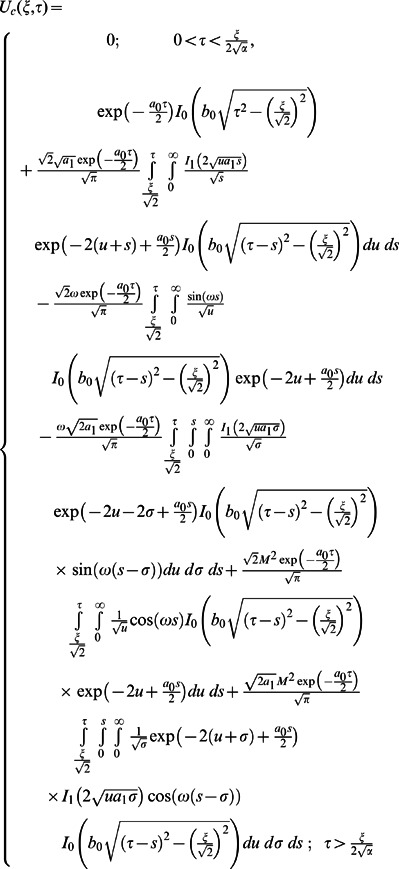
(57)


and
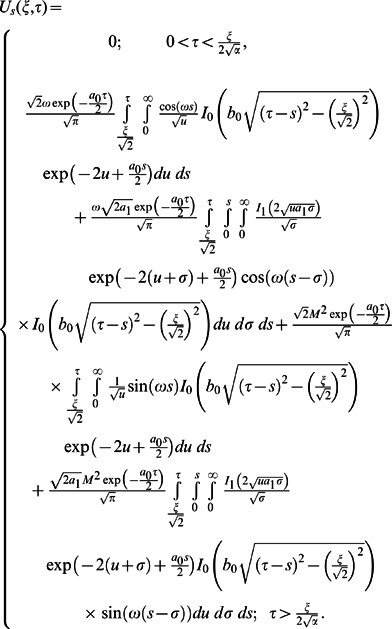
(58)


Equivalent expressions for the velocities 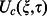
and
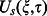
can also be derived from Eqs. 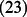
and 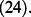
For example, decomposing 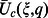
given by equation 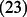
under the form
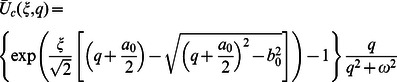


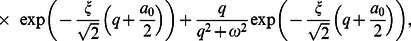



we can write
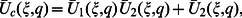
(59)


where
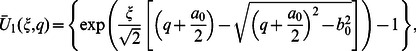
(60)

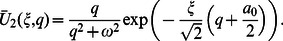
(61)


Applying the inverse Laplace transforms to Eqs.
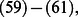
we find that
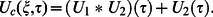
(62)

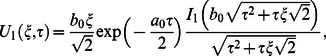
(63)

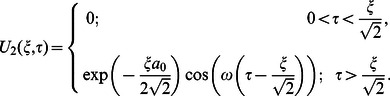
(64)


Consequently, introducing Eqs. (63) and (64) into Eq. (62), we obtain
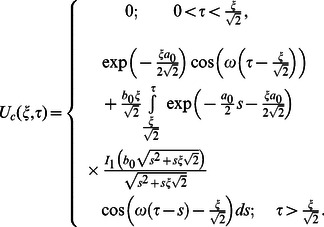
(65)


Following a similar way, we also obtain
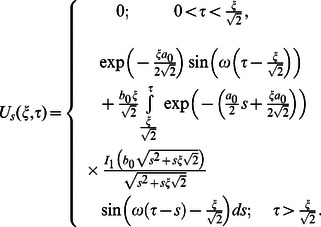
(66)


**Case-II: Solution of the problem for** 
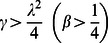


Let us now consider the expressions of velocity fields and tangential stresses when 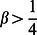. From the system of equations 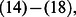
we obtain
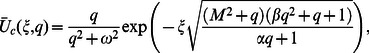
(67)

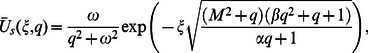
(68)

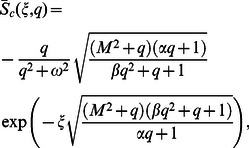
(69)

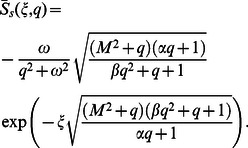
(70)


Here the second grade equation 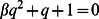 has complex roots.

In order to find the Laplace inverse transform of 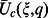, we write Eq. (67) as
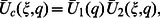
(71)where



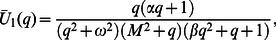
(72)

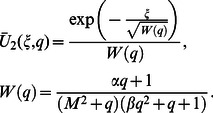
(73)


The Laplace inverse transform of Eq. 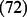
is given by
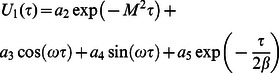


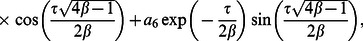
(74)where



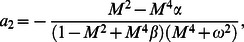


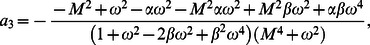


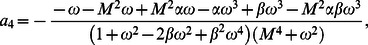


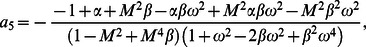


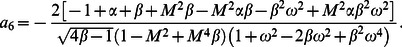
(75)


Moreover, the Laplace inverse transform of Eq. 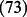
yields
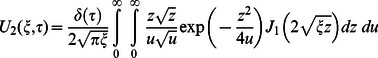


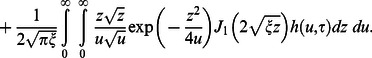
(76)

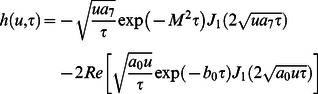


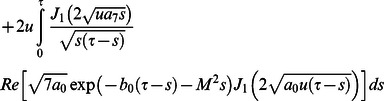


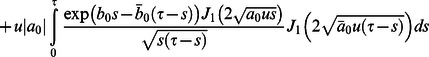


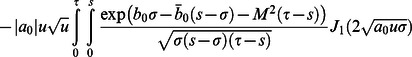


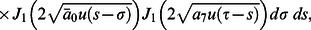
(77)where 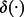
is the Dirac delta function, 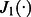
is the Bessel function of the first kind of order one and



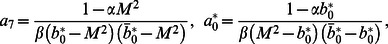


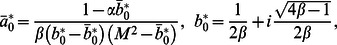


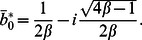
(78)


In view of the relations 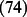
and 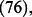
it clearly results
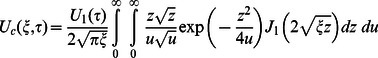


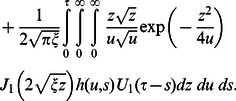
(79)


Adopting a similar procedure for the sine oscillation of the boundary, we get an expression similar to Eq.
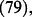
with
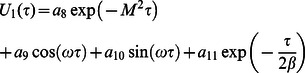


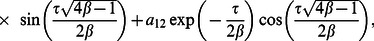
(80)


where
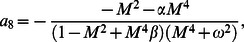


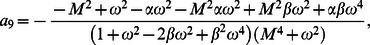


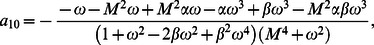


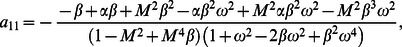


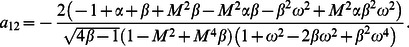
(81)


The corresponding expressions for the shear stresses are given by
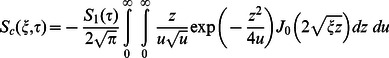


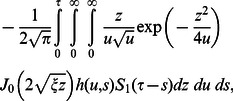
(82)

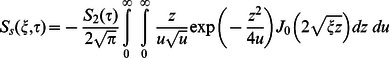


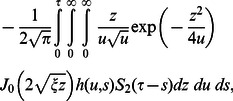
(83)




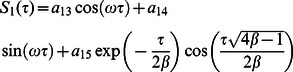


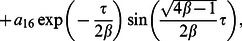
(84)




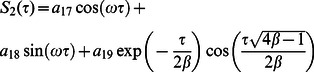
(85)

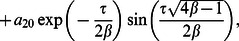


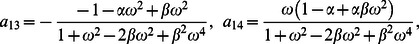





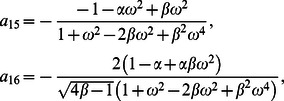


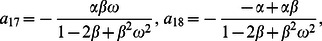


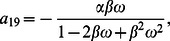


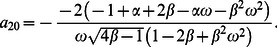
(86)where 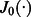
is the Bessel function of the first kind of order zero.

## Limiting Solutions

In this section, for the accuracy of results, we consider a limiting case of our solutions. More exactly, we substitute 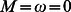
into equations 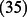
and 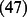
and recover the solutions
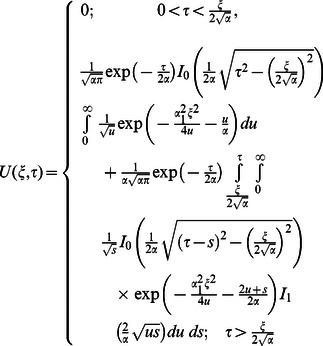
(87)

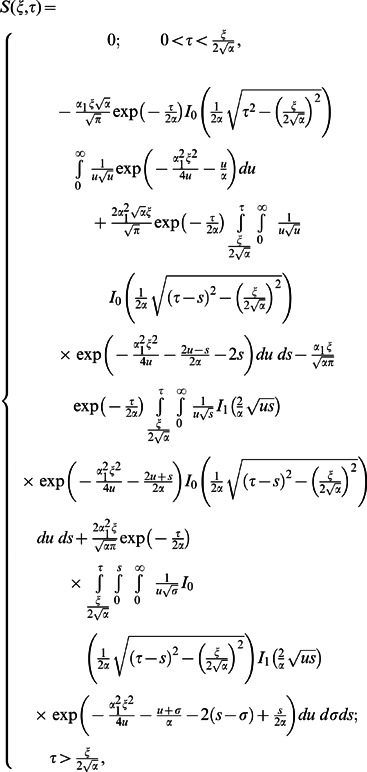
(88)obtained by Vieru et al. [40, Eqs. 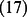
and 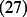]. Similarly, we can also obtained the solutions of Khan et al. [41] from the present solution as special cases by taking the magnetic parameter 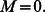 Furthermore, the solutions corresponding to Newtonian and Oldroyd-B fluid also appear as the limiting cases of the present solutions.

## Results and Discussion

**Figure 2:**
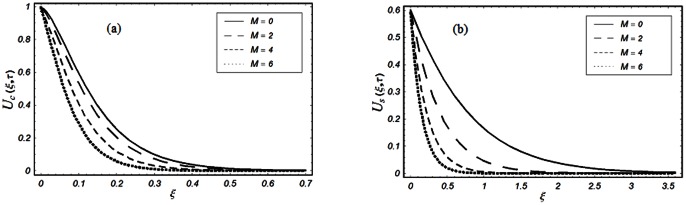
Profiles of the dimensionless velocity corresponding to relations (65) and (66) for different values of 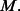

**Figure 3:**
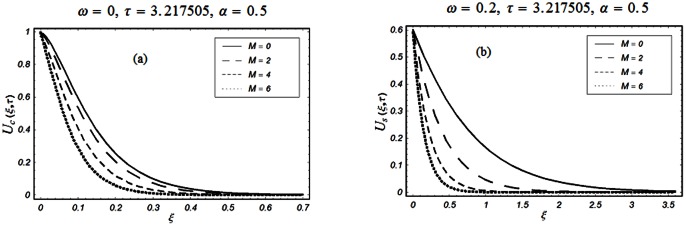
Profiles of the dimensionless velocity corresponding to relations (35) and (36) for different values of 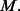

**Figure 4:**
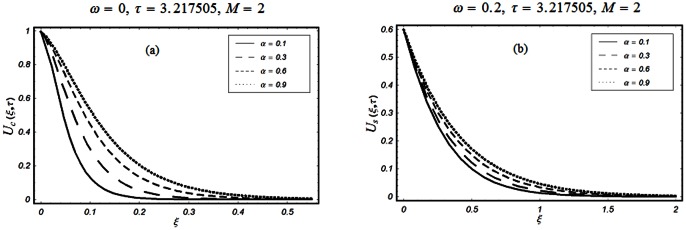
Profiles of the dimensionless velocity corresponding to relations (35) and (36) for different values of 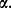

**Figure 5:**
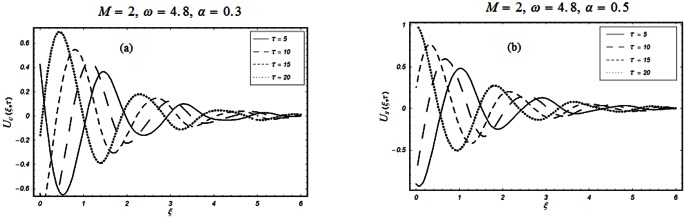
Profiles of the dimensionless velocity corresponding to relations (65) and (66) for different values of 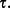

**Figure 6:**
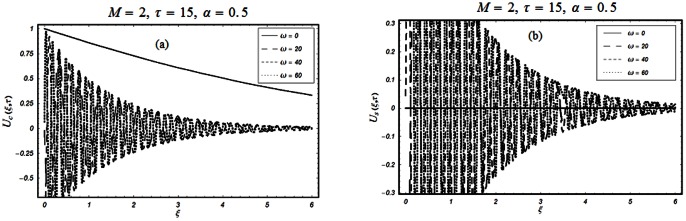
Profiles of the dimensionless velocity corresponding to relations (65) and (66) for different values of 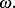

**Figure 7:**
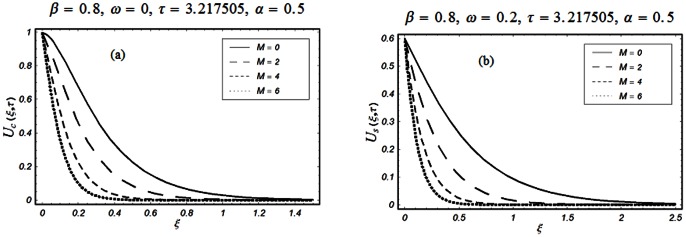
Profiles of the dimensionless velocity corresponding to relations (79) with Eqs. (74) and (80) for different values of 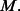.

**Figure 8:**
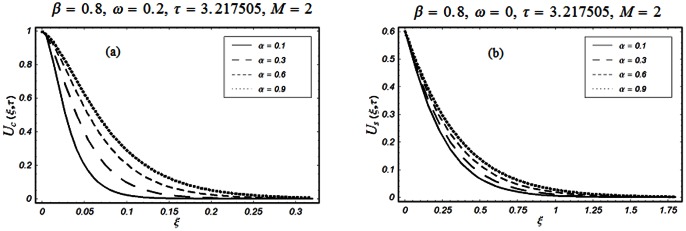
Profiles of the dimensionless velocity corresponding to relations (79) with Eqs. (74) and (80) for different values of 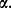.

**Figure 9:**
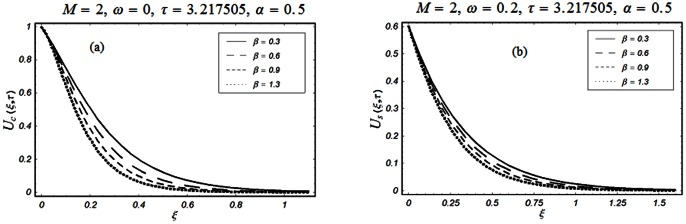
Profiles of the dimensionless velocity corresponding to relations (74) and (80) for different values of 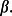

**Figure 10:**
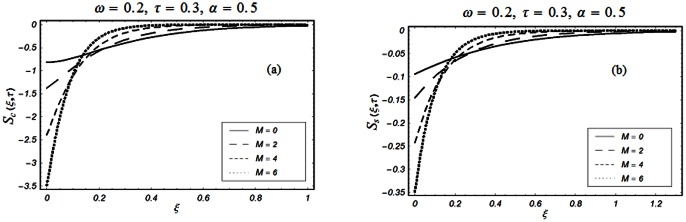
Profiles of the dimensionless shear stress corresponding to relations (55) and (56) for different values of 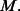

**Figure 11:**
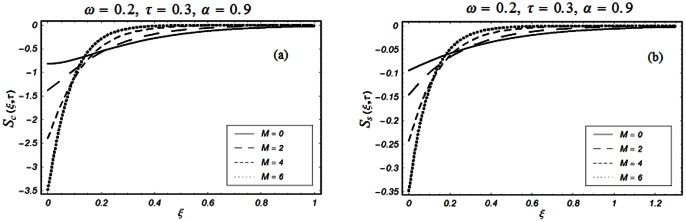
Profiles of the dimensionless shear stress corresponding to relations (47) and (48) for different values of 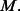

**Figure 12:**
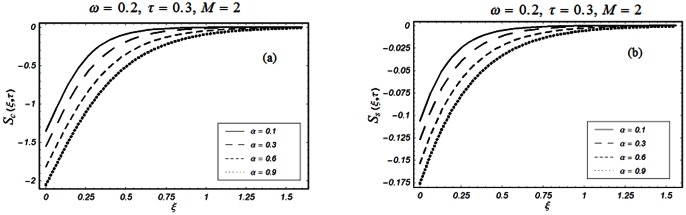
Profiles of the dimensionless shear stress corresponding to relations (47) and (48) for different values of 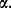

**Figure 13:**
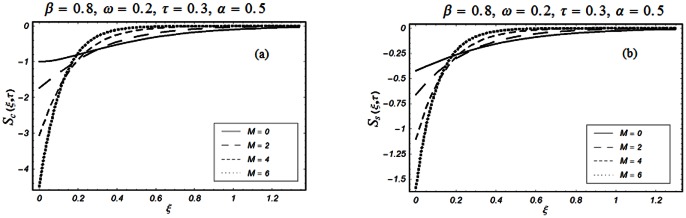
Profiles of the dimensionless shear stress corresponding to relations (82) and (83) for different values of 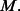

**Figure 14:**
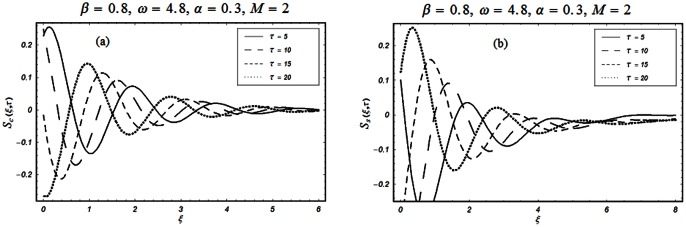
Profiles of the dimensionless shear stress corresponding to relations (82) and (83) for different values of 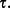

**Figure 15:**
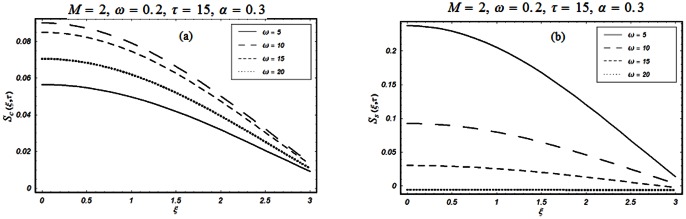
Profiles of the dimensionless shear stress corresponding to relations (82) and (83) for different values of 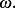

**Figure 16:**
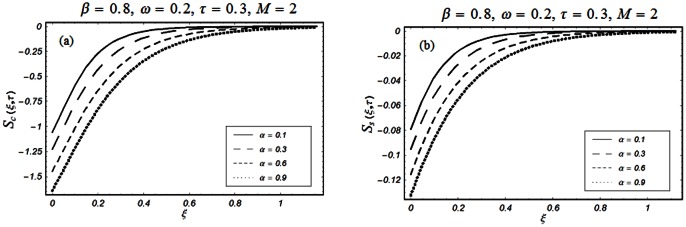
Profiles of the dimensionless shear stress corresponding to relations (82) and (83) for different values of 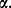

**Figure 17:**
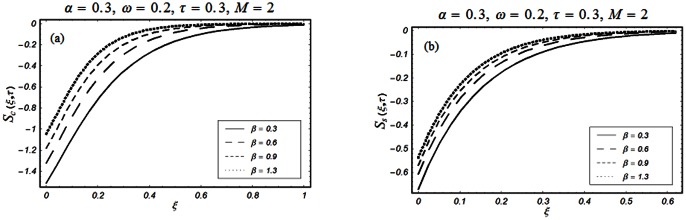
Profiles of the dimensionless shear stress corresponding to relations (82) and (83) for different values of 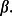

The objective of the present paper is to study the unsteady MHD flow of a Burgers' fluid over an oscillating plate when the relaxation time satisfies the conditions 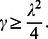
The closed form solutions involve integrals of Bessel functions, terms with only Bessel functions and other integrals are obtained using the Laplace transform technique. These solutions of velocity and shear stress are plotted using the symbolic computational software Mathematica by performing the ordinary numerical integrations. The profiles of velocity fields and shear stresses for both sine and cosine oscillations of the plate are presented in Figs. 2, 3, 4, 5, 6, 7, 8, 9, 10, 11, 12, 13, 14, 15, 16, 17 for different values of the embedded flow parameters. These parameters include the magnetic parameter, also called Hartmann number 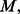
fluid parameters 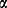
and 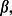
oscillating frequency 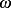
and dimensionless time 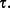


Figs. 2, 3, 4, 5, 6 are drawn so as to show the velocity profiles when the relaxation time satisfies the condition 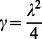 equivalently 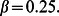 The influence of the Hartmann number 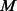
and then of the magnetic field on the fluid motion is shown in Figs. 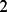
and 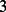
for 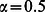
and 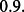
The magnetic field has a significant influence on the velocity field. It is clearly seen from these figures that the velocity of the fluid and the boundary layer thickness decrease if 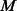
increases for both types of oscillations of the boundary. This is not a surprise as the transverse magnetic field produces a resistance force (Lorentz force) that is similar to the drag force that tends to oppose the flow and to reduce the velocity of the fluid. It is further concluded from the comparison of Figs. 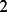
(a) and 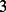
(a) that when 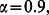
the velocity profiles decay early compare to 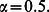
The influence of the parameter 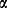
on the velocity profile is shown in Fig. 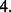
The velocity of the fluid is an increasing function of 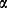
for both types of oscillations of the boundary. However, as expected, for large values of 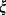
the velocity of the fluid tends to zero.

Figs. 5 & 6 show the periodic nature of the flow. In Fig. 5, the velocity profiles for different values of 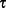
are shown. It is observed that the velocity is developing and fluctuating around zero. For both types of oscillations of the boundary, the velocity has its maximum value at the boundary with gradual decay in its amplitude of oscillation and tends to zero away from the plate. Fig. 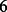 depicts the variation of velocity with oscillating frequency 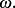
This figure displays the periodic response of the flow to the cosine and sine oscillations of the plate. For 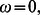
it is clear that the velocity corresponding to the cosine oscillations of the boundary has its maximum value whereas for the sine oscillations it is zero. This fact also results from the imposed boundary conditions 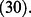
However, for large values of 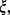
the fluctuation reduces and the velocity approaches zero.

Figs. 7, 8, 9 are displayed for the velocity profile when 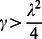 or equivalently 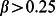
for both the cosine and sine oscillations of the plate. From first two Figs. 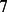 & 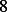, we noticed that the effects of 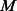
and 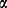
on the velocity profiles are qualitatively similar to those observed in Figs. 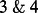
for 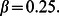
However, these results are different quantitatively. It is further observed from these figures that the velocity profiles decay early for 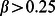
compare to 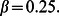
Physically, it is due to the fact that for large values of rheological parameter
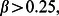
the fluid motion retards and the velocity profiles approaches to zero before than 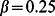
for which the velocity changes are more moderately. Fig. 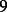
shows the variation of velocity for different values of 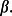
It is found that the velocity and boundary layer thickness decrease when 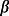
increases. However, it is observed that the decrease in the boundary layer thickness for the cosine oscillations of the plate is more visible than the sine oscillations of the plate.

Figs. 10, 11, 12, 13, 14, 15, 16, 17 are prepared to discuss the variations of the shear stress for both cosine and sine oscillations of the plate. The first three figures (10, 11, 12) are plotted for
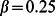
and the last five (13, 14, 15, 16, 17) are displayed for 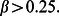
As expected, the behaviors of the velocity and shear stress with respect to 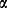
(Figs. 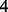
& 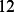
and 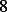
& 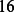), 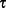
(Figs. 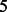 & 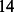), 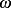
(Figs. 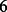 & 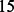) and 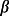
(Figs. 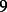 & 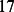) are qualitatively the same. Their behavior with respect to 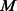
(Figs. 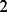 & 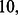
3 & 11 and 7 & 13) are opposite near the plate and the same elsewhere. The velocity of the fluid decreases with respect to 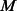
in the whole flow domain while the shear stress increases near the plate and decreases everywhere else.

## Conclusions

In this paper, we have studied the MHD flow of Burgers' fluid when the relaxation time satisfies the conditions 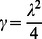 and 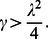
The governing equations are modelled and the closed form solutions are obtained using the Laplace transform technique. The analytical results are displayed graphically and the effects of various emerging flow parameters on the velocity and shear stress are shown. It is found that the magnetic parameter and the rheological fluid parameters have strong influence on the velocity and shear stress fields. It is observed that for large values of rheological parameter
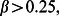
the fluid motion retards and the velocity profiles approaches to zero early than 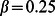
for which the velocity changes are more moderately. Furthermore, these solutions also show the periodic nature of the flow. The existing solutions in the literature are recovered as a special case of the obtained solutions. Hence we are confident at the accuracy of our presented results. For future studies, we have planned to extend this work to the case when the relaxation time satisfies the condition 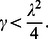 The present problem can also be extended to the MHD flow of Burgers' fluid over a plate embedded in a porous medium. There are several other directions where the present work can be continued.
